# The asymmetric plasma membrane—A composite material combining different functionalities? Balancing Barrier Function and Fluidity for Effective Signaling

**DOI:** 10.1002/bies.202300116

**Published:** 2023-09-15

**Authors:** Gerhard J. Schütz, Georg Pabst

**Affiliations:** ^1^ Institute of Applied Physics TU Wien Vienna Austria; ^2^ Biophysics Institute of Molecular Bioscience (IMB) NAWI Graz University of Graz Graz Austria; ^3^ BioTechMed Graz Graz Austria; ^4^ Field of Excellence BioHealth—University of Graz Graz Austria

**Keywords:** diffusion, fluidity, lipids, membrane asymmetry, permeability, plasma membrane

## Abstract

One persistent puzzle in the life sciences is the asymmetric lipid composition of the cellular plasma membrane: while the exoplasmic leaflet is enriched in lipids carrying predominantly saturated fatty acids, the cytoplasmic leaflet hosts preferentially lipids with (poly‐)unsaturated fatty acids. Given the high energy requirements necessary for cells to maintain this asymmetry, the question naturally arises regarding its inherent benefits. In this paper, we propose asymmetry to represent a potential solution for harmonizing two conflicting requirements for the plasma membrane: first, the need to build a barrier for the uncontrolled influx or efflux of substances; and second, the need to form a fluid and dynamic two‐dimensional substrate for signaling processes. We hence view here the plasma membrane as a composite material, where the exoplasmic leaflet is mainly responsible for the functional integrity of the barrier and the cytoplasmic leaflet for fluidity. We reinforce the validity of the proposed mechanism by presenting quantitative data from the literature, along with multiple examples that bolster our model.

## Graphical abstract

The lipid composition of the two plasma membrane leaflets is highly asymmetric. Here we speculate whether lipid asymmetry reflects nature's need for harmonizing conflicting demands. We assign two plasma membrane functions to the two leaflets: the outer leaflet provides resistance to hydrophilic substances, and the inner leaflet fluidity to signaling molecules.

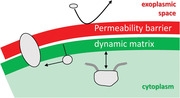

**Abbreviations**
DPPC: dipalmitoyl phosphatidylcholine
LAT: linker for activation of T cells
TCR: T cell receptor
ZAP70: Zeta‐chain‐associated protein kinase 70

## INTRODUCTION

Cellular plasma membranes are highly asymmetric. This statement naturally pertains to membrane proteins,^1^ where it emerges as a consequence of the orientation of the proteins’ transmembrane regions evoked by the asymmetric insertion process in the ER, and by the asymmetry of posttranslational modifications. Also, the more or less transient association of peripheral membrane proteins contributes to the overall asymmetry. The functional relevance of protein asymmetry is apparent, given the different tasks of the cell's exoplasmic versus endoplasmic surface.

The situation becomes less clear‐cut when it comes to lipids, and in fact, a persistent enigma in cell biology revolves around the asymmetry of lipids across the lipid bilayer. But let us take a step back: In fact, it is already remarkable per se that eukaryotic and bacterial cell membranes are lipid bilayers and not lipid monolayers. In the kingdom of life, lipid monolayers do indeed exist in archaea, where bipolar tetraether lipids span the full membrane from the cell inside to the outside.^2^ So why did evolution end up in two types of membrane, a lipid monolayer for most archaea and a lipid bilayer for eukaryotes and bacteria?^3^

In this hypothesis article, we aim to emphasize that lipid bilayers possess a distinctive advantage by conferring vastly distinct physical properties to their two leaflets—a state, which nature realized by means of lipid asymmetry. Proposed already 50 years ago,^4^ it is nowadays well accepted that the outer and inner leaflets differ in their lipid content: while the exoplasmic leaflet hosts preferentially lipids with saturated fatty acyl chains, the cytoplasmic leaflet is composed of lipids containing a variety of (poly‐)unsaturated fatty acids.^5^, ^6^ Besides the plasma membrane of eukaryotic cells, also bacterial membranes were shown to be asymmetric,^7^, ^8^ although fewer details concerning the fatty acid distribution are known. Thus, membrane asymmetry appears to be a hallmark of plasma membranes across eukaryotes and many prokaryotes. The energetic costs for establishing and maintaining plasma membrane asymmetry, however, are substantial: in total, including futile conformational change cycles, flippases, and floppases require an average of tens to hundreds of ATP hydrolysis events to actively transport one lipid molecule across the bilayer.^9^ In this review, we would like to put our focus on the question of potential functional benefits arising from lipid asymmetry across the plasma membrane, which would warrant the energetic costs.

Different concepts have been put forward to motivate the evolutionary benefits of favoring energetically expensive asymmetric plasma membranes over simple symmetric bilayers. These concepts are mostly centered around the charge imbalance of mammalian plasma membranes, and in particular on the outstanding enrichment of phosphatidylserine and phosphatidylinositol in the cytoplasmic leaflet. For example, the predominant location of positively charged residues of integral plasma membrane proteins in the cytosol suggests a co‐alignment with the two major anionic phospholipids, which may assist proper protein orientation.^10^, ^11^, ^12^ Even more so, the transmembrane imbalance of phosphatidylserine—and particularly its reversal, for example, under pathophysiological conditions—is central to numerous signaling processes including apoptosis and blood coagulation.^13^ In addition to membrane potential and surface charge, membrane asymmetry is known to affect various other bulk bilayer properties, including leaflet structure, elasticity, curvature, or overall stability toward membrane‐active compounds.^14^, ^15^, ^16^, ^17^ An example is the redistribution of the cone‐shaped phosphatidylethanolamine, which was observed to regulate the disassembly of the contractile ring in the final stages of cell division.^18^

In this article, however, we try to inspect the question of lipid asymmetry from a different viewpoint. We hypothesize that lipid asymmetry allows plasma membranes to reconcile two seemingly conflicting tasks:

- The plasma membrane needs to be sufficiently impermeable to act as a barrier.
- The plasma membrane needs to be sufficiently fluid to enable dynamical processes such as protein interactions and the spread of membrane‐associated signals.

**FIGURE 1:**
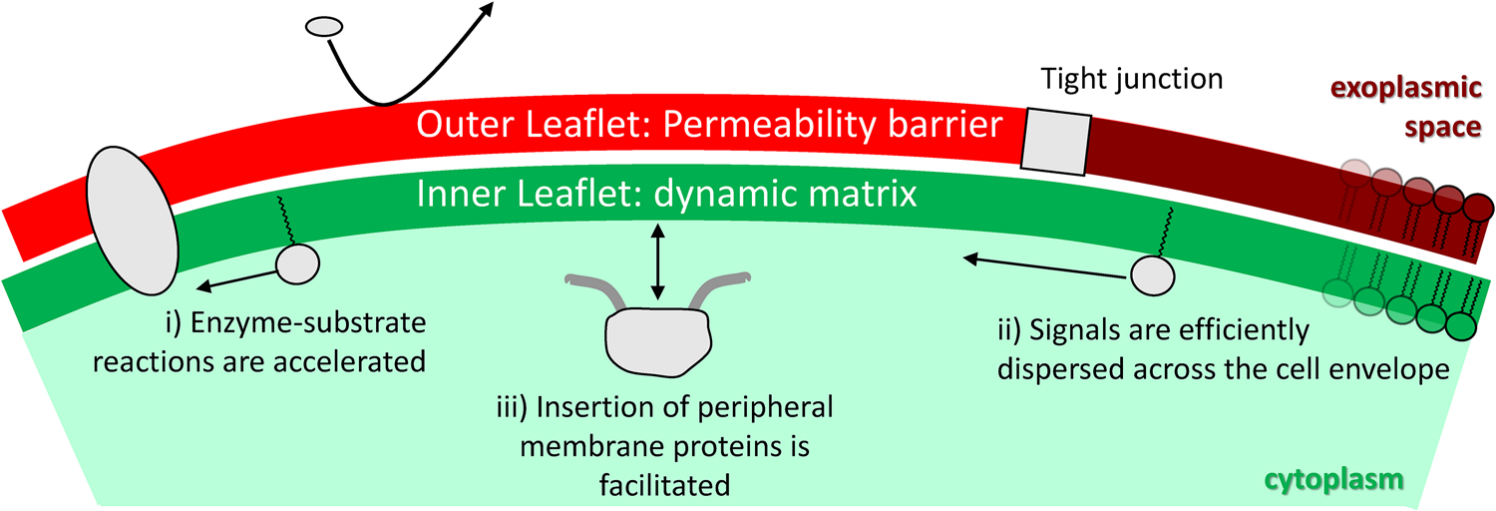
Sketch of the plasma membrane, with the exoplasmic leaflet drawn in red, the cytoplasmic leaflet in green. Dark red and light red indicate basolateral and apical membrane in polarized cells. In our model, the key function of the outer leaflet is constituting a permeability barrier, whereas the fluidity of the inner leaflet is important for forming a dynamic matrix for (i) enzyme‐substrate reaction, (ii) dispersion of signals, and (iii) the association of peripheral membrane proteins or protein domains. See main text for more details.

Our idea is that the two different leaflets act as a composite material combining two different functionalities (Figure 1):

- The outer leaflet provides resistance to hydrophilic substances.
- The inner leaflet provides fluidity to signaling molecules such as inner leaflet lipids and monotopic or peripheral membrane proteins.

The hypothesis is supported by recent experiments employing a fluorescent reporter for membrane packing.^6^ These experiments unveiled a distinct phase state, with the exoplasmic leaflet exhibiting a more ordered phase and the cytoplasmic leaflet displaying a more disordered phase. These findings align remarkably well with the observed compositional asymmetry. In the following, we describe the two major propositions of our hypothesis.

## PROPOSITION 1. THE OUTER LEAFLET OF THE PLASMA MEMBRANE FUNCTIONS AS PERMEABILITY BARRIER

One central functionality of the cellular plasma membrane is to create an efficient barrier to the uncontrolled influx or efflux of small polar molecules. Mathematically, the barrier function is well described by the Meyer‐Overton rule,^19^ where the permeability coefficient is given by $P=\frac{K\cdot D}{d}$ with K being the partition coefficient of the membrane‐permeating molecule between the aqueous and the organic phase, D is the diffusion constant in the membrane, and d is the membrane thickness.

Consistent with $P\propto \frac{1}{d}$, the two bilayer leaflets can be considered to act as two resistances in series,^20^ hence the permeabilities defined as the inverse resistances add reciprocally:

$$\frac{1}{P_{total}}=\frac{1}{P_{o.l.}}+\frac{1}{P_{i.l.}}$$

where *P_total_*, *P*_*o*.*l*._, and *P*_*i*.*l*._ refer to the permeabilities of the total bilayer, the outer leaflet and the inner leaflet, respectively. In case of large permeability differences, the two membrane leaflets act more or less independently from each other,^21^ thus it suffices to discuss the permeability across each leaflet separately.

For the transport of various polar nonionic substances such as water and urea across bilayers, the fluidity was observed to correlate well with permeability for a variety of lipid compositions^22^; an impressive 2000‐fold difference was reported for the water permeability of fluid versus gel‐phase DPPC.^23^ Consistently, when liposomes of composition reflecting the exoplasmic and cytoplasmic plasma membrane leaflet were compared, permeabilities for various polar substances were 18‐ to 90‐fold higher for the cytoplasmic composition.^24^

One specific feature of lipid mixtures mimicking the exoplasmic leaflet is the ability to display coexisting liquid‐ordered and liquid‐disordered domains.^25^ Lipid mixtures mimicking the cytoplasmic leaflet in turn do not display such phenomena, but form a homogenous liquid‐disordered phase.^26^ Liquid‐ordered domains are enriched in cholesterol and saturated lipids, while liquid‐disordered domains contain mostly unsaturated lipids and only about one half to one third of the cholesterol of liquid‐ordered domains. Cholesterol is known to increase the lateral packing of hydrocarbons, that is, decreases the overall area per lipid molecule, which in turn will increase the resistance for membrane‐permeating molecules. Indeed, the permeability of water correlates most strongly with the area per lipid rather than the overall membrane thickness.^27^ The much lower permeability of exoplasmic leaflets can thus be understood by cholesterol‐mediated increased packing of its lipids. The ability to display coexisting domains also implies the formation of domain boundaries which could facilitate the permeability through some defect mechanism. Indeed, molecular dynamics simulations indicated the transit of water or oxygen along local packing defects formed by cholesterol and unsaturated lipids in liquid‐ordered domains.^28^ This leads to a less dramatic but still remarkable seven‐fold permeability difference between the liquid‐ordered and disordered phase.

Concerning the cytoplasmic leaflet, we can further reflect on the dominant contribution of the area per lipid for permeability.^27^ Of note, the lateral area of (poly)unsaturated lipids is known to be larger than that of lipids with saturated hydrocarbons.^29^, ^30^ The abundance of polyunsaturated hydrocarbon chains in the cytoplasmic leaflet of mammalian plasma membranes thus facilitates passive transport. Moreover, the packing of lipid headgroups, and hence the accessibility of hydrophobic region for solutes, was shown to be a dominating factor for water permeability.^27^ Interactions of the lipids in each leaflet with its bordering electrolytic solutions, as well as the hydrogen bonding abilities of the inner leaflet aminophospholipids (phosphatidylethanolamine, phosphatidylserine) is thus likely to additionally modulate the permeability of each leaflet.

Taken together, the permeability of the outer leaflet can be expected to be much smaller than the permeability of the inner leaflet (*P*_*o*.*l*._ ≪ *P*_*i*.*l*._), and the total plasma membrane permeability can thus be well approximated by the outer leaflet permeability *P_total_* ≈ *P*_*o*.*l*._.

## PROPOSITION 2. THE INNER LEAFLET OF THE PLASMA MEMBRANE ENHANCES THE DYNAMICS OF SIGNALING MOLECULES

It is well known that fluorescent lipid analogs and lipid‐anchored proteins show higher diffusion rates in the cytoplasmic compared to the exoplasmic leaflet of the plasma membrane.^6^, ^31^, ^32^ Quantitative studies revealed approximately two‐fold enhanced mobility of lipid‐anchored proteins at the cytoplasmic versus exoplasmic leaflet.^6^ Here, we posit that the evolutionary significance of cellular signaling processes led to the favoring of high fluidity in the cytoplasmic plasma membrane leaflet. This speculation arises from the understanding that fluidity plays a crucial role in facilitating efficient cellular signaling mechanisms. In the following, we highlight the potential benefits of high membrane fluidity:

- Enzyme‐substrate reactions are accelerated. The inner leaflet of the plasma membrane hosts a variety of enzyme‐substrate pairs that are crucial for cellular signaling processes, including kinases, phosphatases, and GTPases such as Ras‐family proteins. Many of these enzymes are monotopic, that is, they do not contain a transmembrane domain but instead are anchored to the cytoplasmic leaflet of the plasma membrane only.^33^ Examples include Src‐family kinases,^34^ G‐proteins,^35^ or Ras GTPases.^36^ As the frequency of diffusion‐mediated encounters in the membrane scales linearly with the diffusion constant,^37^ chemical reactions of these enzymes with their membrane‐bound substrates are strongly accelerated.
- Signals are efficiently dispersed across the cell envelope. Often, the triggering of a few receptor molecules suffices to generate a global intracellular signal. There is therefore a need to efficiently and rapidly amplify the original trigger event. An example is the activation of T cells, where a single antigenic peptide can exert a specific T cell response.^38^ In this case, a critical step is the signal propagation from the triggered T cell receptor (TCR) to downstream signaling molecules such as LAT and SLP‐76.^39^ Upon activation at phosphorylated cytoplasmic chains of the TCR complex, the Syk‐family kinase ZAP70 unbinds from the TCR—but remains associated with the cytoplasmic leaflet of the plasma membrane—and phosphorylates membrane‐anchored LAT after a 2‐dimensional diffusional search.^40^ A second example are polarized cells, where many signals need to be transmitted between basolateral and apical membrane regions. Such plasma membrane regions are formed in monolayers of epithelial or endothelial cells. The separation is achieved by a protein‐rich meshwork encircling the cells termed tight junctions.^41^ Besides stabilizing the intercellular contacts, tight junctions also form efficient diffusion barriers, both to solutes in the exoplasmic space as well as for membrane constituents. Interestingly, signal dispersion is possible for constituents of the cytoplasmic leaflet, whereas outer leaflet lipids cannot cross tight junctions without being translocated to the inner leaflet.^42^, ^43^ The cytoplasmic leaflet can hence be regarded as a continuous fluid matrix for lipids and peripheral proteins, which spans across tight junctions and couples the basolateral and apical membranes.
- Insertion of peripheral membrane proteins is facilitated. In general, packing defects, as they occur in membranes enriched in unsaturated fatty acids, facilitate the targeting of proteins carrying membrane anchoring motifs such as amphipathic helices or alkyl chains.^44^, ^45^ Compositional asymmetry, additionally, likely results in differential membrane tension, with one leaflet being compressed, the second leaflet being stretched.^46^ Notably, similar effects can be achieved by packing different amounts of the same lipid in each leaflet (number asymmetry). While the net curvature across flat membrane regions cancels out, the opposing lateral stress affects the mechanical properties of the two leaflets: the compressed leaflet gets stiffened, whereas the stretched leaflet shows loosened lipid packing.^47^ Assuming increased tensile stress in the cytoplasmic leaflet, this would further increase the targeting of peripheral membrane proteins.^48^, ^49^ Indeed, the cytoplasmic leaflet is the target for the (transient) association of a manifold of peripheral membrane proteins.^50^, ^51^ By enabling the shuttling of proteins between the plasma membrane and the cytoplasm, cells accomplish a functional connection between these compartments. But also cytoplasmic domains of integral membrane proteins—for example the CD3 co‐receptors of the T cell receptor complex—show affinity to the cytoplasmic leaflet of the plasma membrane,^52^, ^53^, ^54^ thereby modulating the associated signaling events.^55^

## DISCUSSION

The main point of this hypothesis article is to highlight a potential link between lipid asymmetry across the eukaryotic plasma membrane and two of the membrane's main functional tasks, impermeability and fluidity. Obviously, this approach suffers from oversimplifying the inherent complexity of the system. Indeed, the advent of protocols for the fabrication of artificial lipid‐only asymmetric membranes has spurred fundamental biophysical studies on their specific properties,^14^ giving rise to a variety of new insights that complement our proposition.

In particular, we largely ignored here the coupling of the two membrane leaflets. For example, the different lateral stresses stored in each leaflet can affect membrane elasticity,^46^ which is additionally modulated by the ability of cholesterol to rapidly translocate across the bilayer.^56^ Astonishingly rigid asymmetric membranes were reported independently from several groups.^57^, ^58^, ^59^ In these cases, the elasticity of asymmetric membranes was much lower than that of symmetric membranes composed of the same lipids and symmetric membranes formed from either of their leaflets.^59^

Also, the fluidity and lipid packing of one leaflet were observed to affect the opposing leaflet. The enrichment of highly chain‐asymmetric lipids in one leaflet can slow down the lateral diffusion of lipids in the other leaflet,^60^ or cholesterol‐containing lipid domains in one leaflet are apt to induce highly ordered domains in leaflets that would otherwise just form a homogenous phase.^61^, ^62^ Further, the extent of hydrocarbon chain interdigitation can increase or decrease the packing density of lipids in the opposing leaflet.^63^ It is still discussed controversially, however, to what extent such data of lipid‐only membranes can be transferred to the live cell plasma membrane. Of note, although lipids typical for the cytoplasmic leaflet do not show phase separation when studied in model bilayers,^26^ the cortical actin cytoskeleton was observed to induce the formation of more ordered domains^64^ in cells. Consistently, differential partitioning of protein anchors in phase‐separated model membranes correlated well with the differential localization of the same anchors in cell membranes, indicating the presence of tunable signaling domains in the plasma membrane.^65^ Together, it appears plausible that each membrane protein possesses its characteristic nanoenvironment,^66^ which modulates its mobility and conformational flexibility. In this context we would like to refer the reader to the rich literature on the presence of membrane domains in the plasma membrane (for reviews see^67^, ^68^, ^69^, ^70^) and discussions on the controversy in this field.^71^, ^72^, ^73^

While we are just on the verge of revealing the biophysics pertaining to the function of asymmetric membranes, including the coupling to integral proteins,^74^ it is clear that their behavior is much richer than those of symmetric membranes. How much do we expect to fail in our discussion by not considering contributions from transbilayer coupling mechanism described above? Indeed, such scenarios might tweak the properties of exoplasmic or cytoplasmic leaflets to some extent. However, the overall big picture still remains valid: plasma membranes apparently combine an outer leaflet physical barrier against permeation with a highly efficient fluid signaling “platform.” Local variations in protein and lipid composition, including lipid scrambling then may add additional local functions to plasma membranes in response to external or cytosolic stimuli. These may include, for example, exo‐/endocytosis, or the transient formation of signaling complexes, but might also extend to larger length scale and affect overall cell motility or deformability, all of which are matter of ongoing research efforts.

## CONFLICT OF INTEREST STATEMENT

The authors do not have any conflict of interest.

## Data Availability

Data sharing is not applicable to this article as no new data were created or analyzed in this study.
